# The Efficiency of Convalescent Plasma Therapy in the Management of Critically Ill Patients Infected With COVID-19: A Matched Cohort Study

**DOI:** 10.3389/fmed.2022.822821

**Published:** 2022-06-16

**Authors:** Chun Pan, Hui Chen, Jianfeng Xie, Yingzi Huang, Yi Yang, Bin Du, Haibo Qiu

**Affiliations:** ^1^Jiangsu Provincial Key Laboratory of Critical Care Medicine, Department of Critical Care Medicine, School of Medicine, Zhongda Hospital, Southeast University, Nanjing, China; ^2^Department of Critical Care Medicine, The First Affiliated Hospital of Soochow University, Soochow University, Jiangsu, China; ^3^Medical Intensive Care Unit, Peking Union Medical College Hospital, Peking Union Medical College and Chinese Academy of Medical Sciences, Beijing, China

**Keywords:** convalescent plasma therapy, mechanical ventilation, viral shedding, mortality, COVID-19

## Abstract

**Background:**

The convalescent plasma of patients who recover from coronavirus disease 2019 (COVID-19) contains high titers of neutralizing antibodies, which has potential effects on the viral shedding of severe acute respiratory syndrome coronavirus 2 (SARS-CoV-2) and improving the prognosis of patients with COVID-19. The goal of this study was to clarify the effects of convalescent plasma therapy on the 60-day mortality and negative conversion rate of SARS-CoV-2 during the hospitalization of patients with severe and life-threatening COVID-19 infection.

**Methods:**

This was a retrospective, case-matched cohort study that involved patients with severe COVID-19 infections. The patients who received convalescent plasma therapy were matched by age, sex, diabetes, hypertension, heart failure, the onset of symptoms to hospital admission, respiratory support pattern, lymphocyte count, troponin, Sequential organ failure assessment (SOFA), glucocorticoid, and antiviral agents to no more than three patients with COVID-19 who did not receive convalescent plasma therapy. A Cox regression model and competing risk analysis were used to evaluate the effects of convalescent plasma therapy on these patients.

**Results:**

Twenty-six patients were in the convalescent plasma therapy group, and 78 patients were in the control group. Demographic characteristics were similar in both groups, except for the SOFA score. Convalescent plasma therapy did not improve 60-day mortality [hazard ratio (HR) 1.44, 95% CI 0.82–2.51, *p* = 0.20], but the SARS-CoV-2 negative conversion rate for 60 days after admission was higher in the convalescent plasma group (26.9 vs. 65.4%, *p* = 0.002) than in the control. Then, a competing risk analysis was performed, which considered events of interest (the negative conversion rate of SARS-CoV-2) and competing events (death) in the same model. Convalescent plasma therapy improved events of interest (*p* = 0.0002).

**Conclusion:**

Convalescent plasma therapy could improve the SARS-CoV-2 negative conversion rate but could not improve 60-day mortality in patients with severe and life-threatening COVID-19 infection.

**Clinical Trial Number:**

The study was registered at ClinicalTrials.gov (NCT04616976).

## Introduction

The coronavirus disease 2019 (COVID-19) has infected more than 494 million people, and 6 million people have died as a result of the disease thus far (1). There was a poor prognosis for patients with severe and life-threatening COVID-19 infection. There are several treatment therapies for COVID-19, and some novel oral antivirals could reduce mortality or hospitalization rates and adverse events among COVID-19 patients; however, there was not enough evidence of the use of antiviral agents associated with significant clinical benefits in patients with severe and life-threatening COVID-19 (2–6).

Convalescent plasma has a high titer of neutralizing antibodies and has potential effects on viral clearance. Convalescent plasma therapy was recommended as a treatment protocol for COVID-19 by WHO (7). The first study of convalescent plasma therapy involved the use of convalescent plasma to treat 5 patients with COVID-19. Following the transfusion of convalescent plasma, clinical status was improved, viral loads were decreased, and neutralizing antibody titers were increased (8). The recent five clinical trials that involved outpatients showed conflicting results of convalescent plasma therapy. A double-blind randomized controlled trials (RCTs) in Argentina recruited 160 older outpatients (aged ≥ 75 years) at risk for disease progression, and COVID-19 convalescent plasma could reduce disease progression (16 vs. 31%, *p* < 0.05) (9). In addition, another double-blind RCT in the USA confirmed the results and found that COVID-19 convalescent plasma administration led to a reduction in hospitalization within 28 days (2·9 vs. 6·3%; *p* = 0·004) (10). However, the other studies did not report the benefit of convalescent plasma for the patients (11, 12).

To date, the omicron variant of severe acute respiratory syndrome coronavirus 2 (SARS-CoV-2) has high transmissibility, and the early studies indicated lower severity of infection than that of the delta variant. An individual-level data from England found that when compared with delta, omicron had a lower hazard ratio (HR) of hospital admission and death (13). However, the intensive care unit (ICU) admission of the omicron variant of SARS-CoV-2 was still high at 4.6%, mechanical ventilation usage was 1.9%, and the mortality in the hospital was as high as 4.1%. The patients at risk of severe or life-threatening cases of COVID-19, early convalescent plasma therapy (<3 days from symptom onset), and high immunoglobulin G (IgG) titer could improve mortality (14). However, there is not enough evidence to prove the benefits of late-phase convalescent plasma therapy in patients with severe and life-threatening COVID-19 infection.

This study is aimed to clarify the effects of convalescent plasma therapy on 60-day mortality and the SARS-CoV-2 negative conversion rate during the hospitalization of patients with severe and life-threatening COVID-19 infection.

## Materials and Methods

We performed a retrospective-matched cohort study that was conducted in 19 designated hospitals for COVID-19 in Wuhan (Hubei Province) and Huangshi (Hubei Province). The study was approved by the local Research Ethics Board. Consent was waived by the Ethics Commission because of the outbreak of COVID-19. This study was registered on www.clinicaltrials.gov (NCT04616976).

### Study Population

All adult patients with COVID-19 who were admitted to intensive care units (ICUs) of the participating hospitals between 1 January 2020 and 29 February 2020 were screened. The inclusion criteria were as follows: (1) >18 years of age; (2) laboratory-confirmed diagnosis of COVID-19; and (3) respiratory failure requiring advanced respiratory support [i.e., non-invasive mechanical ventilation (NIV), high-flow nasal cannula (HFNC), and invasive mechanical ventilation (IMV)].

We used the definitions of severe and life-threatening COVID-19 infection provided by a former study (15). Severe COVID-19 infection was defined as respiratory distress (≥30 breaths/min; in the resting state, oxygen saturation of 93% or less on room air; or arterial partial pressure of oxygen (PaO_2_)/fraction of inspired oxygen (FIO_2_) of 300 mmHg or less). Life-threatening COVID-19 infection was defined as respiratory failure requiring mechanical ventilation, shock, or other organ failures (apart from the lung) requiring ICU monitoring.

Convalescent plasma was donated by the patients who were fully recovered from COVID-19 in 2 weeks. Convalescent plasma from patients who recovered from COVID-19 was collected and processed *via* plasmapheresis at the Wuhan Blood Center. The plasma products were prepared as fresh-frozen plasma, and the spike protein receptor-binding domain (S-RBD)-specific IgG antibody titer was higher than 640 for convalescent plasma therapy.

### Data Collection

Demographic characteristics, history of comorbidities, vital signs, and laboratory examinations within the first 24 h after hospital admission were extracted from medical records. Treatment and outcome data were also recorded. Sequential organ failure assessment (SOFA) scores were calculated to assess the severity of illness by using data from admission to the ICU. The main exposure of interest was the administration of a dose of at least 200 ml of convalescent plasma therapy. All data were collected by using a case report form.

### Matched Cohort and Outcomes

The main exposure of interest was the administration of convalescent plasma. To ensure that an adequate number of patients who did not receive convalescent plasma, every 20 patients who did not receive convalescent plasma were initially matched to each exposed patient. Matching was performed according to age, sex, hypertension, heart failure, diabetes, symptom onset to hospital admission, respiratory support pattern, lymphocyte count, troponin, SOFA, glucocorticoid, and antiviral agents. Furthermore, the unexposed comparison group was created by assigning each patient who received convalescent plasma to no more than three individuals who did not receive convalescent plasma after matching.

The primary outcome of the study was 60-day mortality. The secondary outcome was the negative conversion rate of SARS-CoV-2 at 60 days.

### Statistical Analysis

Mean ± SD was used to describe the normal distribution. According to the results of Levene's test of homogeneity of variance, *t*-test or corrected *t-*test was used to compare the mean between two groups. Data with a non-normal distribution are described by the median (P25 and P75), and median comparisons between groups were performed by the Mann–Whitney test. Categorical variables were described by percentage and frequency, and comparisons between groups were performed by χ^2^, continuously corrected χ^2^, and Fisher's exact probability test.

We first used a Cox regression model to characterize the relationship between convalescent plasma therapy and 60-day mortality. Based on prior knowledge, baseline variables were selected into the multivariate Cox proportional hazards regression model and included age, sex, hypertension, heart failure, diabetes, symptom onset to hospital admission, respiratory support pattern, lymphocyte count, troponin, SOFA, glucocorticoid, and antiviral agents. To avoid bias induced by missing data, we used multiple imputation by chained equation (MICE) to account for the missing data. Considering that the time to initial convalescent plasma therapy varied, we then treated convalescent plasma therapy as a time-dependent variable in an extended multivariate Cox regression model. Subgroup analyses according to age, sex, diabetes, hypertension, SOFA score, and IMV were performed.

A proportion of patients did not have SARS-CoV-2 RNA clearance until death, and the Cox hazards model is not satisfactory to characterize the relationship between convalescent plasma therapy and the negative conversion rate of SARS-CoV-2. We then performed a competing risk analysis using the Fine and Gray model, which considered events of interest (SARS-CoV-2 RNA clearance) and competing events (death) in the same model.

Values of *p* were calculated to evaluate the differences between groups, and *p* < 0.05 was considered statistically significant. All statistical analyses were performed using R Studio (version 1.2.5019).

## Results

### Analysis Sample

**Figure 1:**
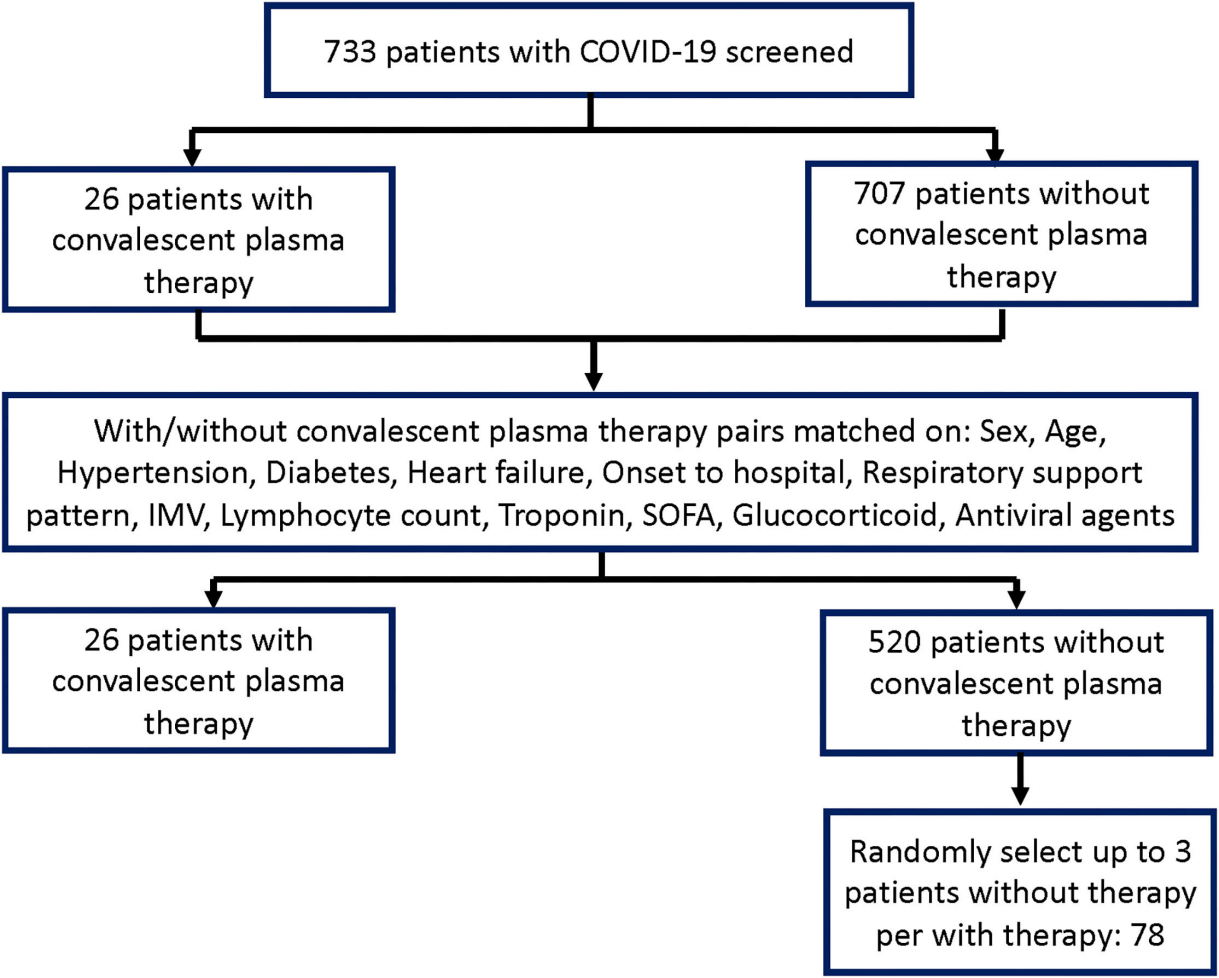
Flow diagram.

The study involved 733 patients with COVID-19 during the study period; 26 critically ill patients had convalescent plasma therapy and were matched at an approximate 1:20 ratio to 520 patients without convalescent plasma therapy. Up to three available patients who did not receive convalescent plasma therapy per patient who did receive convalescent plasma therapy were then chosen at random, resulting in 78 patients who were recruited but did not receive convalescent plasma therapy. Figure 1 depicts the derivation of the sample group for the analyses.

### Patients' Characteristics

Patients' demographic variables are given in Table 1. The age of the groups was similar [63 (59.3, 74.5) vs. 67 (59.0, 71.0), *p* = 0.97], percent of men in the plasma treatment group was 73.1 and 65% in the control group, history of comorbidities included hypertension, chronic cardiac dysfunction, and diabetes and was not different between the two groups. The lymphocyte count at baseline was similar [0.51 (0.35, 0.76) vs. 0.62 (0.39, 0.87), *p* = 0.37], and the percentage of antiviral therapy was similar (76.9 vs. 75.6%, *p* = 1.0). The percentage of steroid use was 38.5% in the convalescent plasma therapy group and 42.3% in the control group (*p* = 0.26). The SOFA score was higher in the convalescent plasma group than in the control group [4 (3. 7.75) vs. 3 (2, 5), *p* = 0.006] (Table 1).

**Table 1:** Characteristics of patients with coronavirus disease 2019 (COVID-19).

**Variables**	**Total (*n* = 104)**	**Convalescent plasma group** **(*n* = 26)**	**Control Group** **(*n* = 78)**	***p***
Age (years) (median, Q1, Q3)	66.5 (59.0, 72.3)	63 (59.3, 74.5)	67 (59.0, 71.0)	0.97
Male (%)	70 (67)	19 (73.1)	51 (65)	0.63
Underlying Disease, *n* (%)	
Hypertension	46 (44)	13 (50)	33 (42)	0.65
Diabetes	16 (15)	4 (15.4)	12 (15.4)	1.0
Chronic cardiac dysfunction	6 (6)	3 (11.5)	3 (4)	0.16
SOFA score (median, Q1, Q3)	3 (3, 5)	4 (3, 7.75)	3 (2, 5)	0.006
Respiratory support (%)				0.09
HFNC/NIV	52 (50)	9 (34.6)	43 (55.1)	
IMV	46 (44.2)	14 (53.8)	32 (41.0)	
ECMO	6 (5.8)	3 (11.5)	3 (3.9)	
Lymphocyte count at admission (×10^9^/L) (median, Q1, Q3)	0.52 (0.39, 0.82)	0.51 (0.35, 0.76)	0.53 (0.42, 0.86)	0.57
Troponin (μg/mL)	26.6 (9.52, 80.47)	39.0 (17.5, 42.5)	23.5 (8.18, 87.8)	0.38
Anti-viral treatment usage, *n* (%)	79 (77)	20 (76.9)	59 (75.6)	1.0
Steroid usage, n (%)	43 (41)	10 (38.5)	33 (42.3)	0.91
Onset to hospital (days) (median, Q1, Q3)	12 (7, 16)	11 (7, 15)	13 (8.5, 19.75)	0.22
Negative conversion rate (%)				0.002
Positive	18 (17)	2 (8)	18 (17)	
Negative	38 (37)	17 (65)	38 (37)	
Dead	48 (46)	7 (27)	48 (46)	
Negative conversion time (median, Q1, Q3)	15 (8.92, 40.67)	26.5 (12.75, 42.67)	14 (8, 27.25)	0.073
60-day mortality (%)	45 (43.3)	12 (46.2)	47 (60.3)	0.30
Hospital Stay (days)	27 (11, 60)	60 (25.75, 60)	19.5 (10, 60)	0.027

*SOFA score, sequential organ failure assessment scores; HFNC, high flow nasal cannular; NIV, non-invasive ventilation; IMV, invasive mechanical ventilation; ECMO, extracorporeal membrane oxygenation*.

### Outcomes

Convalescent plasma therapy did not improve the 60-day mortality of patients with severe or life-threatening COVID-19 infection (46.2 vs. 60.2%, *p* = 0.304). The negative conversion rate of SARS-CoV-2 60 days after admission was higher in the convalescent plasma group (26.9 vs. 65.4%, *p* = 0.002).

#### Convalescent Plasma Therapy Did Not Improve the Mortality of Patients With Severe and Life-Threatening COVID-19 Infection

In the multivariate Cox proportional hazards regression model, after adjusting for age, sex, hypertension, heart failure, diabetes, onset to hospital, respiratory support pattern, lymphocyte count, troponin, SOFA, glucocorticoid, and antiviral agents, convalescent plasma therapy was associated with a reduced 60-day mortality in patients with severe and life-threatening COVID-19 infection (convalescent plasma therapy: HR 0.34, 95% CI 0.17–0.68, *p* = 0.02; Table 2).

**Table 2:** Multivariate Cox proportional hazards regression model to explore convalescent plasma therapy associated with 60-day mortality in severe and life-threatening coronavirus disease 2019 (COVID-19).

**Risk factors**	**HR**	**95%CI**		***p***
Convalescent plasma therapy	0.34	0.17	0.68	<0.01
Sex	0.89	0.47	1.67	0.72
Age	1.03	1.0	1.05	0.03
Hypertension	0.87	0.50	1.54	0.64
Diabetes	0.76	0.36	1.60	0.47
Chronic cardiac dysfunction	0.39	0.09	1.7	0.21
Onset to hospital	0.99	0.96	1.02	0.51
Respiratory support pattern	2.5	1.65	3.88	<0.01
SOFA score	1.06	0.97	1.17	0.19

*SOFA score, sequential organ failure assessment scores*.

However, when convalescent plasma therapy was treated as a time-dependent variable in an extended multivariate Cox regression model, convalescent plasma therapy did not influence 60-day mortality (convalescent plasma therapy: HR 1.44, 95% CI 0.82–2.51, *p* = 0.20; Table 3).

**Table 3:** Extended Cox model to explore effects of convalescent plasma therapy with 60-day mortality in severe and life-threatening coronavirus disease 2019 (COVID-19).

**Risk factors**	**Adjusted HR**	**Adjusted 95%CI**		***p***
Convalescent plasma therapy	1.44	0.82	2.51	0.20
Sex	0.92	0.47	1.79	0.80
Age	1.02	1.0	1.05	0.09
Hypertension	1.12	0.52	2.40	0.78
Diabetes	0.70	0.28	1.71	0.43
Chronic cardiac dysfunction	0.30	0.05	1.78	0.19
Onset to hospital	0.98	0.94	1.02	0.22
Respiratory support pattern	4.09	2.56	6.56	<0.01
SOFA score	0.93	0.83	1.05	0.24

*SOFA score, sequential organ failure assessment scores*.

**Figure 2:**
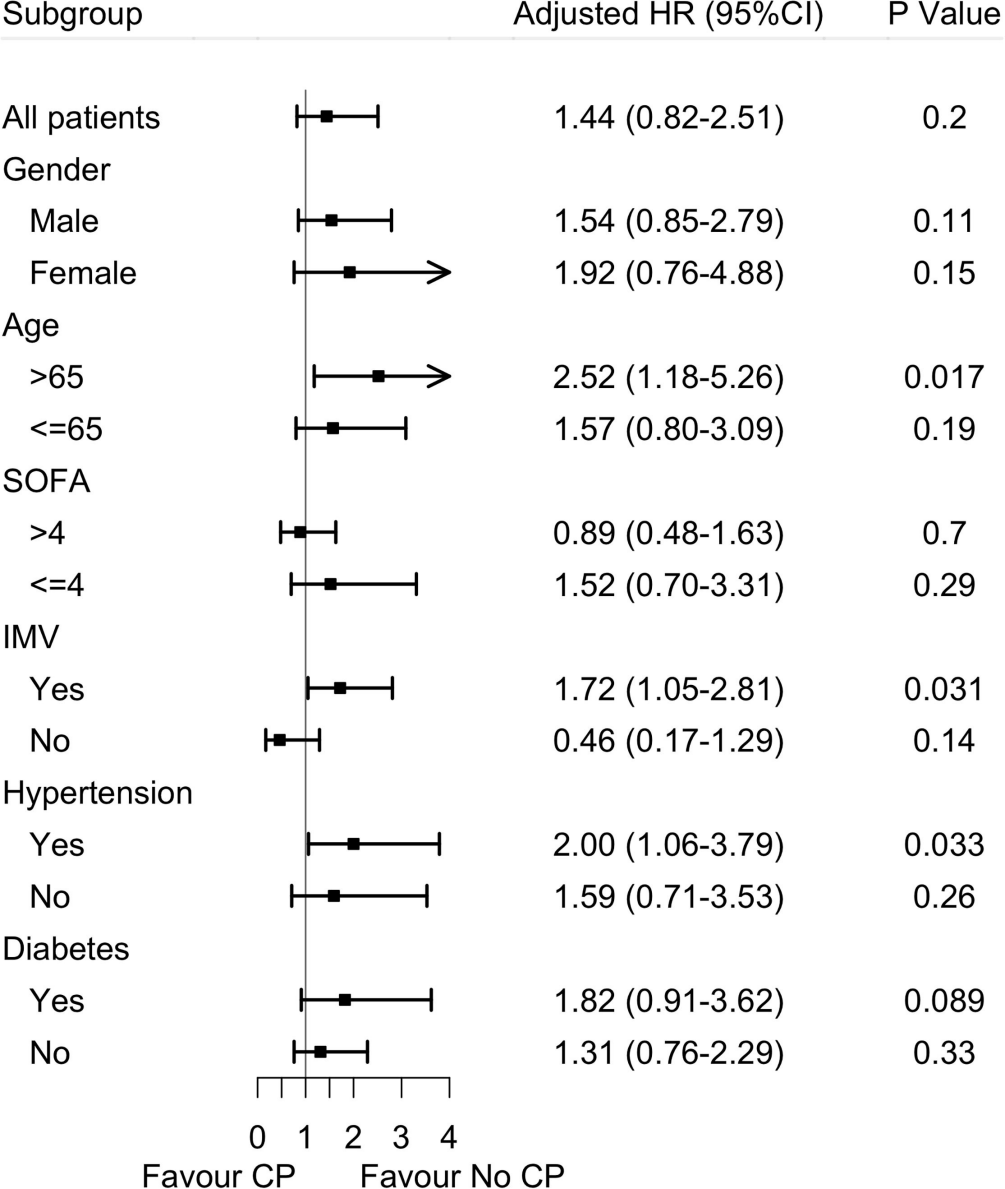
Effects of convalescent plasma therapy on 60-day mortality according to subgroups in severe and life-threatening coronavirus disease 2019 (COVID-19). SOFA score, sequential organ failure assessment scores; IMV, invasive mechanical ventilation; CP, convalescent plasma therapy.

In the subgroup analysis, the effects of convalescent plasma therapy vs. control therapy on 60-day mortality were significantly different across subgroups of older patients, patients receiving IMV, and patients with hypertension (Table 4 and Figure 2).

**Table 4:** Effects of convalescent plasma therapy on 60-day mortality according to subgroups in severe and life-threatening coronavirus disease 2019 (COVID-19).

**Subgroup**	**Adjusted HR**	**95%CI**		***p***
Sex				
Male	1.54	0.85	2.79	0.11
Female	1.92	0.76	4.88	0.15
Age				
>65	2.53	2.28	5.36	0.02
≤ 65	1.57	0.80	3.09	0.19
SOFA score				
>4	0.89	0.48	1.63	0.70
≤ 4	1.52	0.70	3.31	0.29
Hypertension				
Yes	2.00	1.06	3.79	0.03
No	1.59	0.71	3.53	0.26
Diabetes				
Yes	1.82	0.91	3.63	0.09
No	1.31	0.76	2.29	0.33
Invasive mechanical ventilation				
Yes	1.72	1.05	2.81	0.03
No	0.46	0.17	1.29	0.14

*SOFA score, sequential organ failure assessment scores*.

#### Convalescent Plasma Therapy could Improve the Negative Conversion Rate of SARS-CoV-2

**Figure 3:**
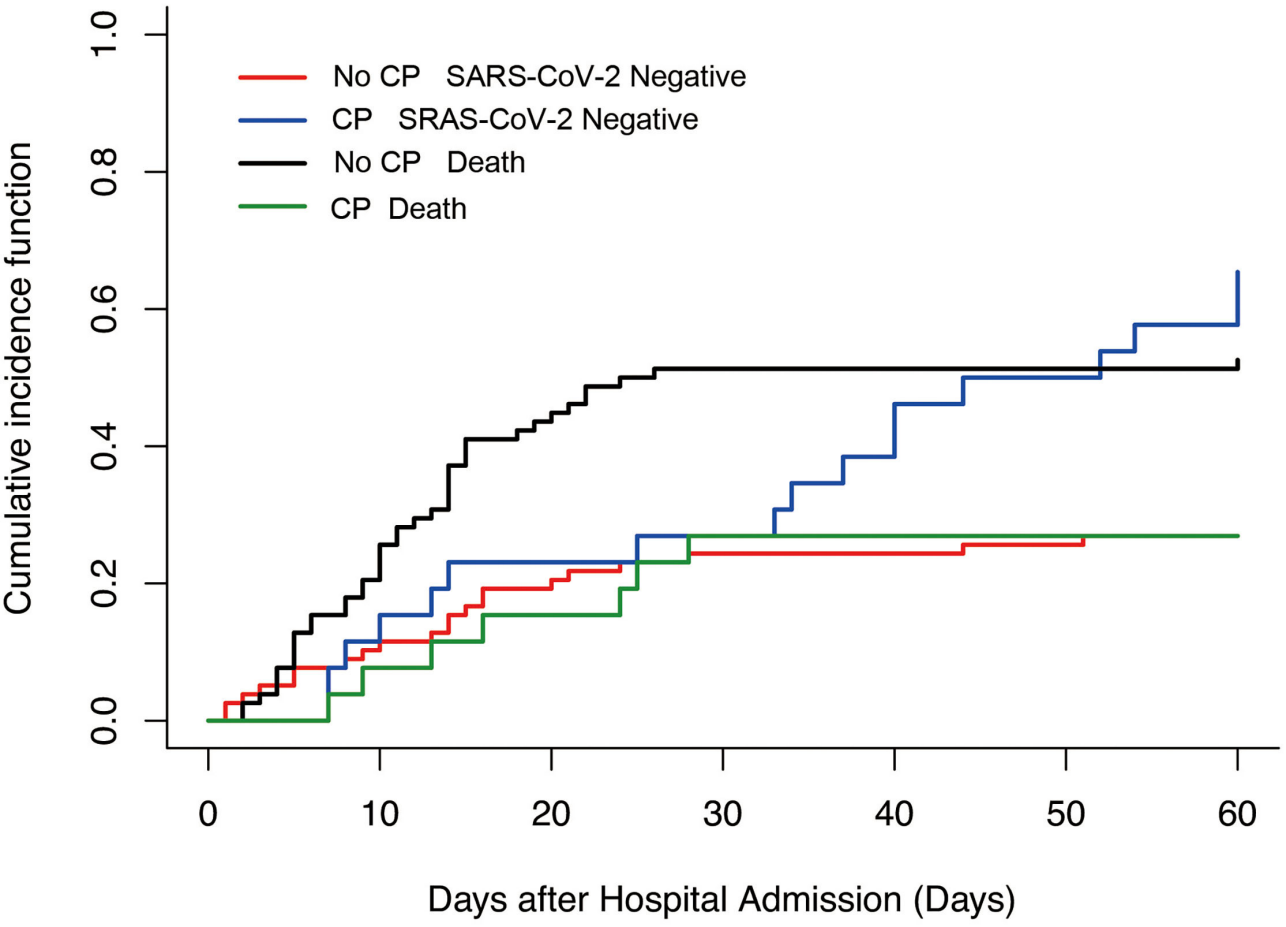
Competing risk analysis to explore Convalescent plasma therapy associated with events of interest in severe and life-threatening coronavirus disease 2019 (COVID-19). No CP SARS-CoV-2 negative vs. CP SARS-CoV-2 negative, *p* = 0.002; No CP death vs. CP death, *p* = 0.02. CP, convalescent plasma therapy.

Because a proportion of patients did not have SARS-CoV-2 RNA clearance until death and the Cox hazards model was not satisfactory to characterize the relationship between convalescent plasma therapy and SARS-CoV-2 negative conversion rate, we then performed a competing risk analysis, which considered events of interest (SARS-CoV-2 negative conversion rate) and competing events (death) in the same model. We found that convalescent plasma therapy improved the SARS-CoV-2 negative conversion rate (convalescent plasma therapy: HR 4.93, 95% CI 2.16–11.23, *p* = 0.0002; Table 5 and Figure 3).

**Table 5:** Competing risk analysis to explore convalescent plasma therapy associated with events of interest in severe and life-threatening coronavirus disease 2019 (COVID-19).

**Risk factors**	**HR**	**95%CI**		***p***
Convalescent plasma therapy	4.93	2.16	11.23	<0.01
Sex	0.92	0.44	1.92	0.83
Age	1.02	0.99	1.04	0.14
Hypertension	0.87	0.38	1.97	0.74
Diabetes	0.31	0.09	1.06	0.062
Chronic cardiac dysfunction	1.86	0.36	9.66	0.46
Onset to Hospital	1.01	0.96	1.05	0.81
Respiratory support pattern	1.25	0.69	2.28	0.46
SOFA score	0.86	0.70	1.07	0.18

*SOFA score, sequential organ failure assessment scores*.

## Discussion

The findings of this study are that in patients with severe and life-threatening COVID-19 infection, convalescent plasma administration could not improve 60-day mortality, but the therapy could improve the virus-negative conversion rate.

High viral loads are associated with the severity and prognosis of viral diseases. In patients with SARS and Middle East respiratory syndrome (MERS) infections, viral loads in patients with severe infection were higher than those in patients with mild infection (16, 17), and patients with a severe infection also had more prolonged viral shedding in respiratory secretions for as long as 21 days (16). In patients with COVID-19, those with severe infection had a longer duration of viral shedding in respiratory samples than those with mild infection [21 (14, 30) days vs. 14 (10–21) days, *p* = 0.04], and the viral loads of respiratory samples of patients with mild infection were peaked in the second week, whereas the viral load of patients with severe infection could be high for as long as 3 weeks from symptom onset (18, 19). Therefore, accelerating virus clearance is the key treatment for COVID-19.

Convalescent plasma therapy could increase SARS-CoV-2-specific neutralizing antibody titers and promote viral shedding in patients with COVID-19. For COVID-19-induced acute respiratory distress syndrome (ARDS), convalescent plasma therapy could decrease viral loads, improve viral shedding within 12 days, and increase SARS-CoV-2-specific neutralizing antibody titers (40–60 before and 80–320 on Day 7) (8). In our study, the median SARS-CoV-2-negative conversion time after convalescent plasma treatment was 14 (8, 27.25) days. Convalescent plasma therapy could increase and maintain the high level of neutralizing antibodies (20); however, a recent multicenter randomized study showed that convalescent plasma therapy did not improve the clinical symptoms of patients with COVID-19 regardless of the disease severity (11). The clinical improvement of patients with severe disease occurred in 91.3% (21/23) vs. 68.2% (15/22) (*p* = 0.03), but for patients with life-threatening COVID-19 infection, clinical improvement was occurred in 20.7% (6/29) vs. 24.1% (7/29) (*p* = 0.83) (15). Our study was consistent with a previous study, and convalescent plasma therapy improved viral shedding in patients with severe and life-threatening COVID-19 infection.

The recent clinical trials involved outpatients showed conflicting results of convalescent plasma therapy. Two studies showed that convalescent plasma therapy reduced disease progression and hospitalization, but the other studies did not find that convalescent plasma was not shown to be efficacious (9–12). The reasons may be related to the timing of convalescent plasma therapy, the underestimation of sample size, and Fc integrity, which has been strongly implicated in COVID-19 convalescent plasma efficacy (21).

However, convalescent plasma therapy could not improve the mortality of patients with severe or life-threatening COVID-19 infection. In a recent study with the US Convalescent Plasma Expanded Access Program (EAP) program, patients with severe or life-threatening infection treated with convalescent plasma were recruited. This study found that mortality was related to the time of plasma transfusion and COVID-19 diagnosis and IgG antibody levels in transfused plasma (14). However, in RCTs, the early use of convalescent plasma therapy with a high neutralizing antibody titer in patients with severe COVID-19 infection did not reduce mortality or improve other clinical outcomes at Day 30 when compared with placebo (22). Our study results were consistent with the results of this study. In our study, we also found that convalescent plasma therapy could not improve 60-day mortality for patients with severe and life-threatening infections. There are several reasons that should be clarified. First, lung and other organ injuries were more severe in these patients, and clearance of SARS-CoV-2 *per se* could not reverse lung and organ injuries at that stage. Second, convalescent plasma therapy was not initiated at an early stage. In our study, therapy was initiated at 17 (5.3, 29.5) days after hospital admission because there was not enough convalescent plasma available at an early stage in Wuhan, China. However, our study clarified that convalescent plasma therapy was safe and well-tolerated.

Elderly patients may benefit from convalescent plasma therapy. In a RCT from Argentina involving 160 older outpatients (mean age, 77.2 years) with mild symptoms who received high titer convalescent plasma within 72 h after the onset of symptoms, the risk of respiratory failure was lower in the convalescent plasma group than in the placebo group (9). Our study found that convalescent plasma therapy could improve the prognosis of older (>65 years) patients. Consistent with this study, older patients may have a lower inflammatory response, and clearance of SARS-CoV-2 could help to improve the syndrome, but this still needs further study for clarification.

During the pandemic of COVID-19, there are many SARS-CoV-2 variants emerged, and most of these variants emerged after convalescent plasma trials were finished. The question is whether convalescent plasma collected during former SARS-CoV-2 variant-infected patients is still effective against current SARS-CoV-2 variants? There are some studies that found the newly emerged variants could escape convalescent plasma neutralization (23, 24). However, the polyclonal nature of convalescent plasma and its derived polyclonal antiserum formulation preserved potential effects on different variants (25).

There are several limitations in this study: first, this is a small case-matched and retrospective study; second, viral loads and SARS-CoV-2-specific neutralizing antibody titers after convalescent plasma therapy of recruited patients were not monitored; third, all patients were treated with several other medications, although we used a retrospective-matched method to eliminate the bias of baseline, and it is difficult to determine whether the improvement was related to therapies other than convalescent plasma.

## Conclusion

In the retrospective-matched cohort study, convalescent plasma therapy improved the SARS-CoV-2 negative conversion rate but did not improve 60-day mortality in patients with severe and life-threatening COVID-19 infection. The results require evaluation in future clinical trials.

## Data Availability Statement

The data analyzed in this study is subject to the following licenses/restrictions: We could not supply the dataset to the others according to stipulation of our country. Requests to access these datasets should be directed to panchun1982@gmail.com.

## Ethics Statement

The studies involving human participants were reviewed and approved by the Local Research Ethics Board. Consent was waived by the Ethics Commission because of the outbreak of COVID-19. The Ethics Committee waived the requirement of written informed consent for participation.

## Author Contributions

HQ and BD designed the study. HQ and YY support this study. CP, HC, and JX selected the data and analysis the data. CP, YH, and HC draw the draft. All authors contributed to the article and approved the submitted version.

## Funding

This work was funded by Jiangsu Provincial Special Program of Medical Science (BE2018743 and BE2019749), the National Science and Technology Major Project for Control and Prevention of Major Infectious Diseases of China (2017ZX10103004), the National Natural Science Foundation of China (81571847 and 81930058), Jiangsu Provincial Key Laboratory of Critical Care Medicine, Department of Critical Care Medicine, Zhongda Hospital, School of Medicine, Southeast University, Grants from the Ministry of Science and Technology of the People's Republic of China (2020YFC0843700), the Open Project of Key Laboratory of Environmental Medical Engineering Ministry of Education (2020EME001), and Key Project of Medical Scientific Research Project of Jiangsu Provincial Health Commission (ZD2021057).

## Conflict of Interest

The authors declare that the research was conducted in the absence of any commercial or financial relationships that could be construed as a potential conflict of interest.

## Publisher's Note

All claims expressed in this article are solely those of the authors and do not necessarily represent those of their affiliated organizations, or those of the publisher, the editors and the reviewers. Any product that may be evaluated in this article, or claim that may be made by its manufacturer, is not guaranteed or endorsed by the publisher.
